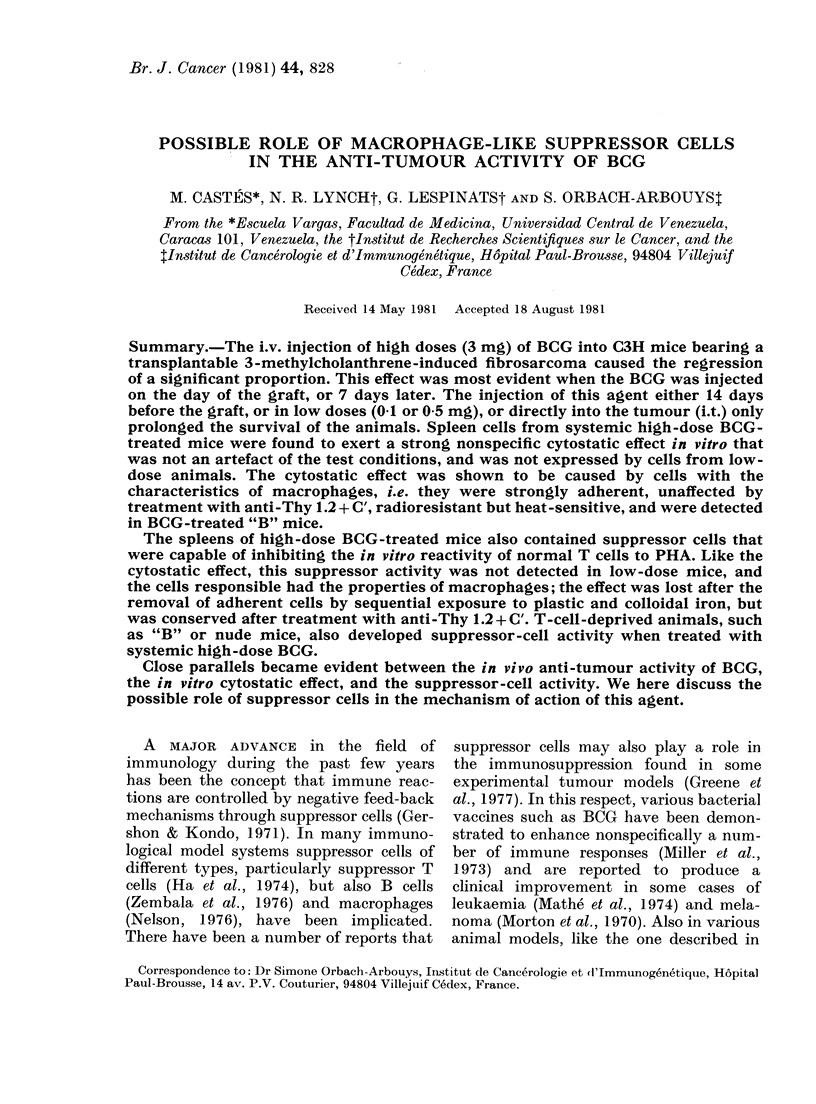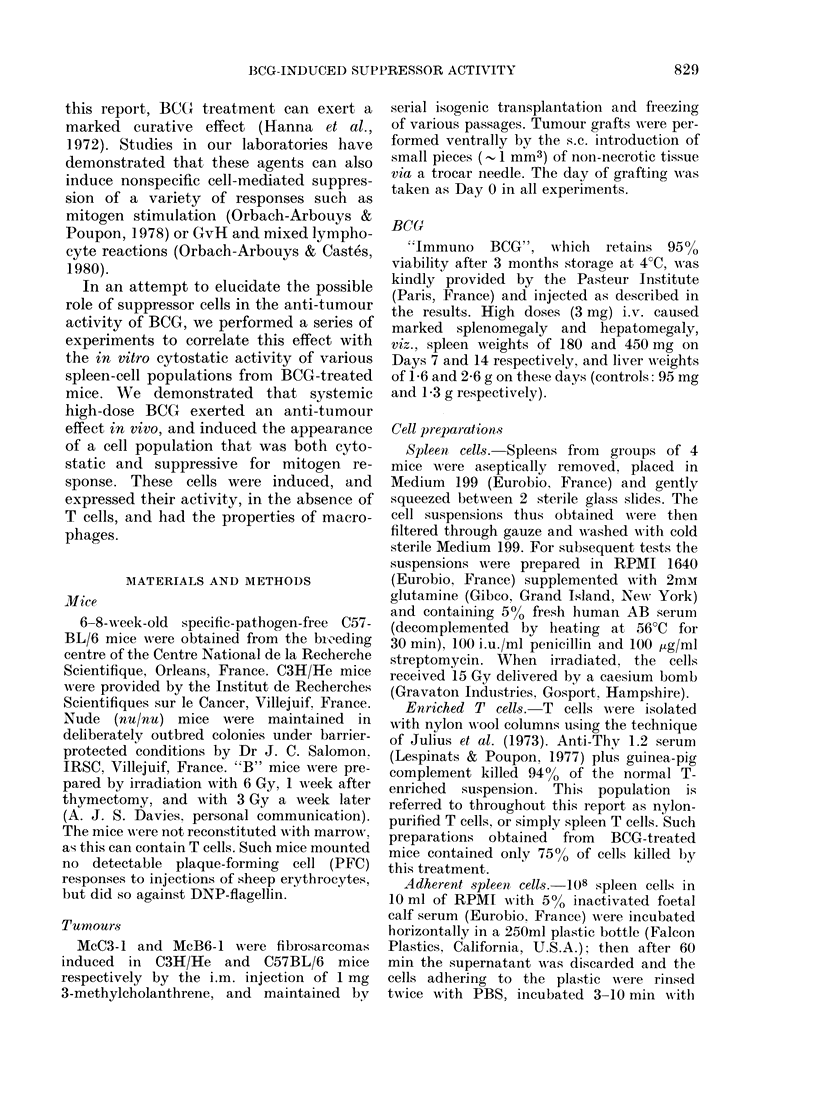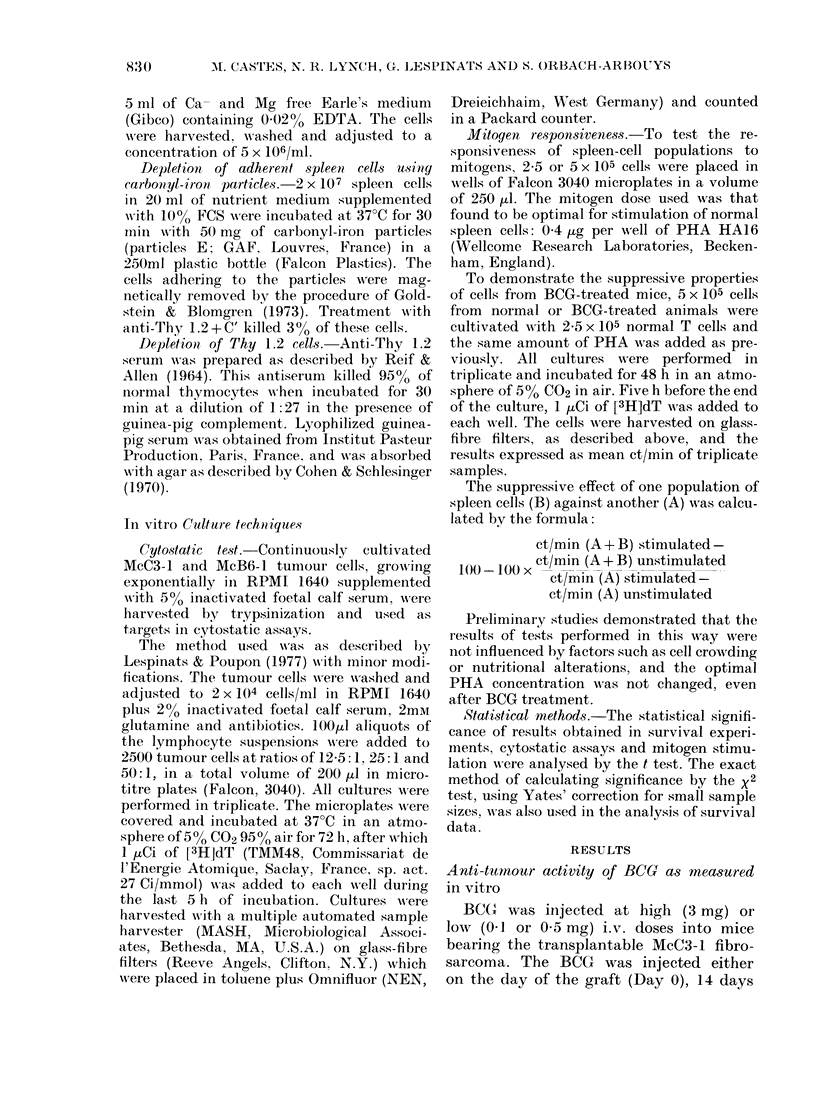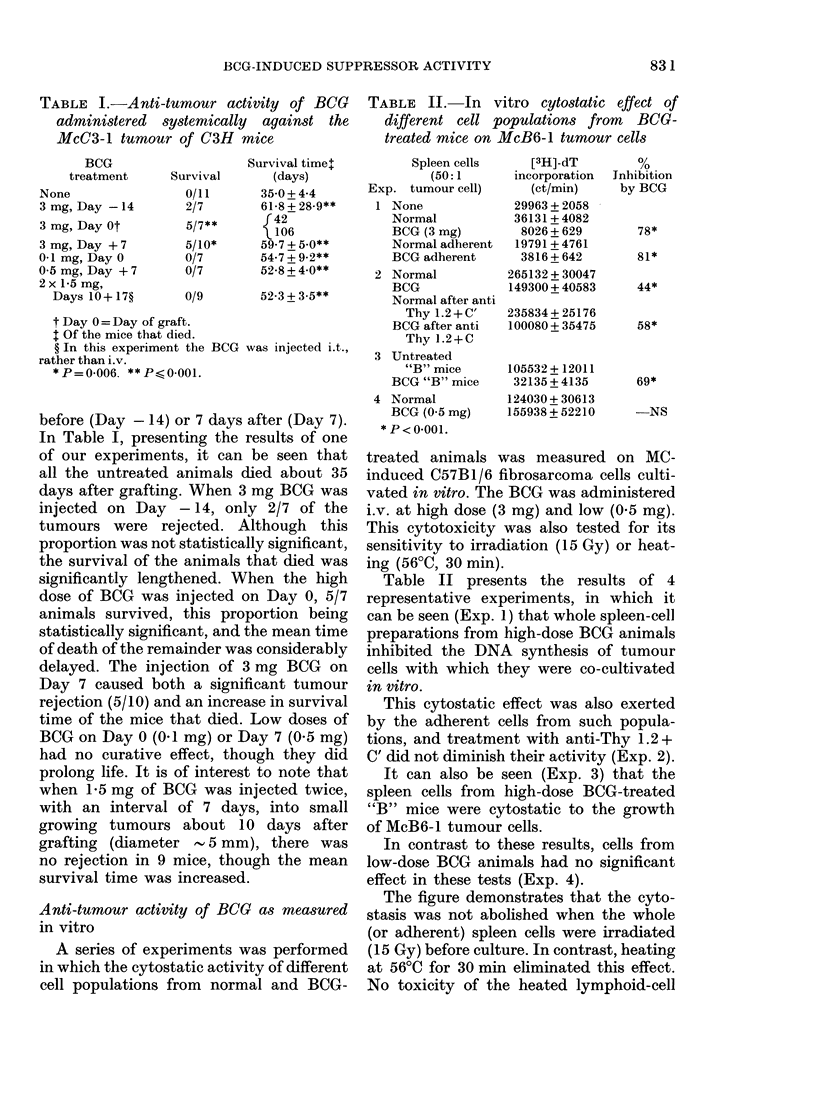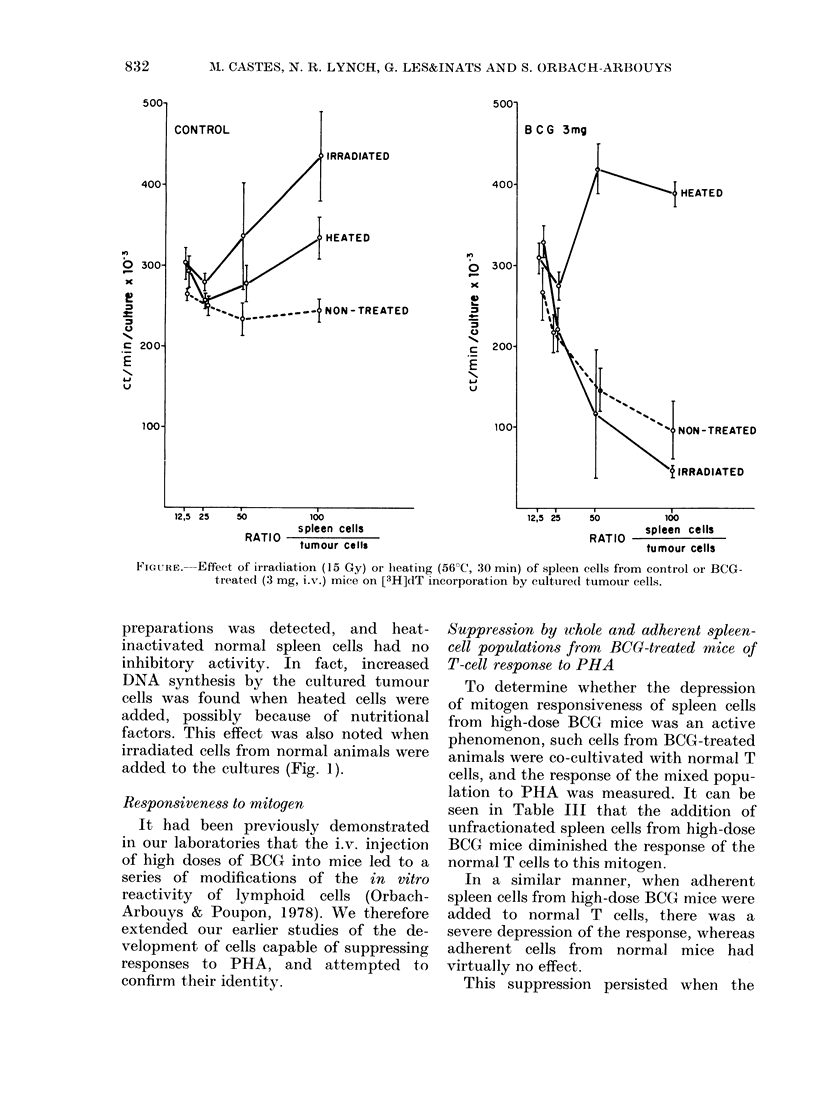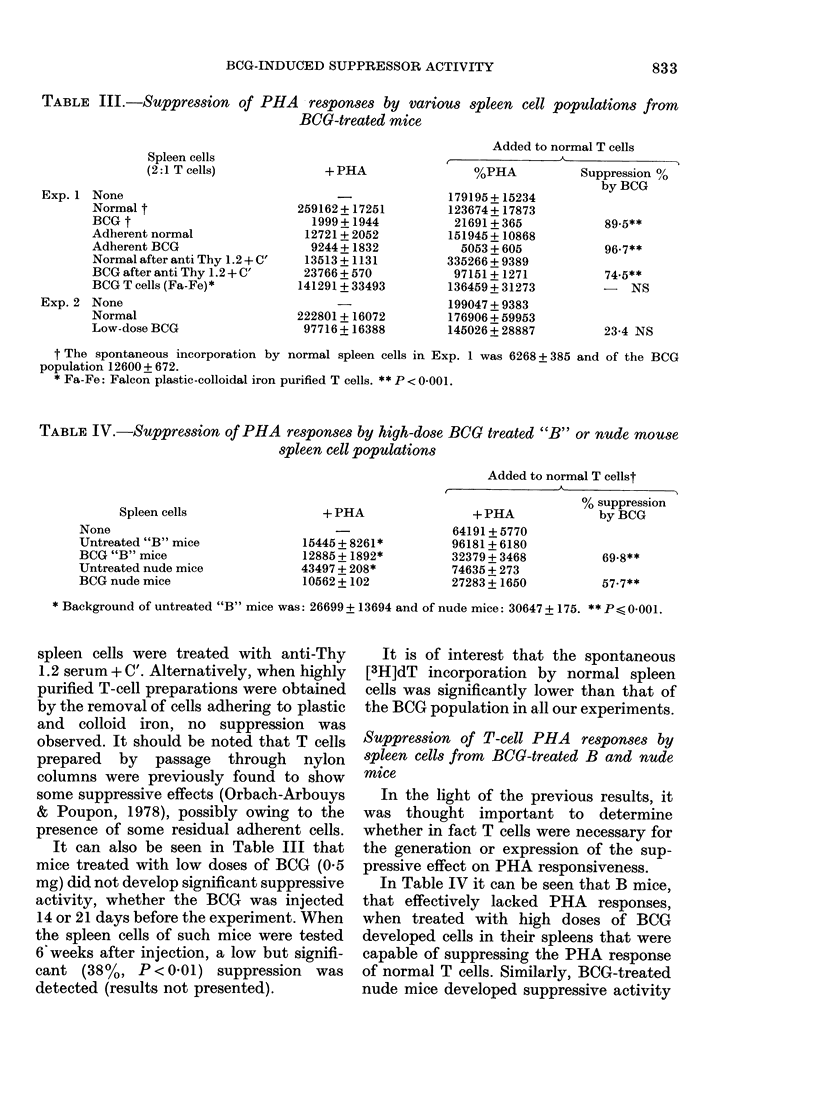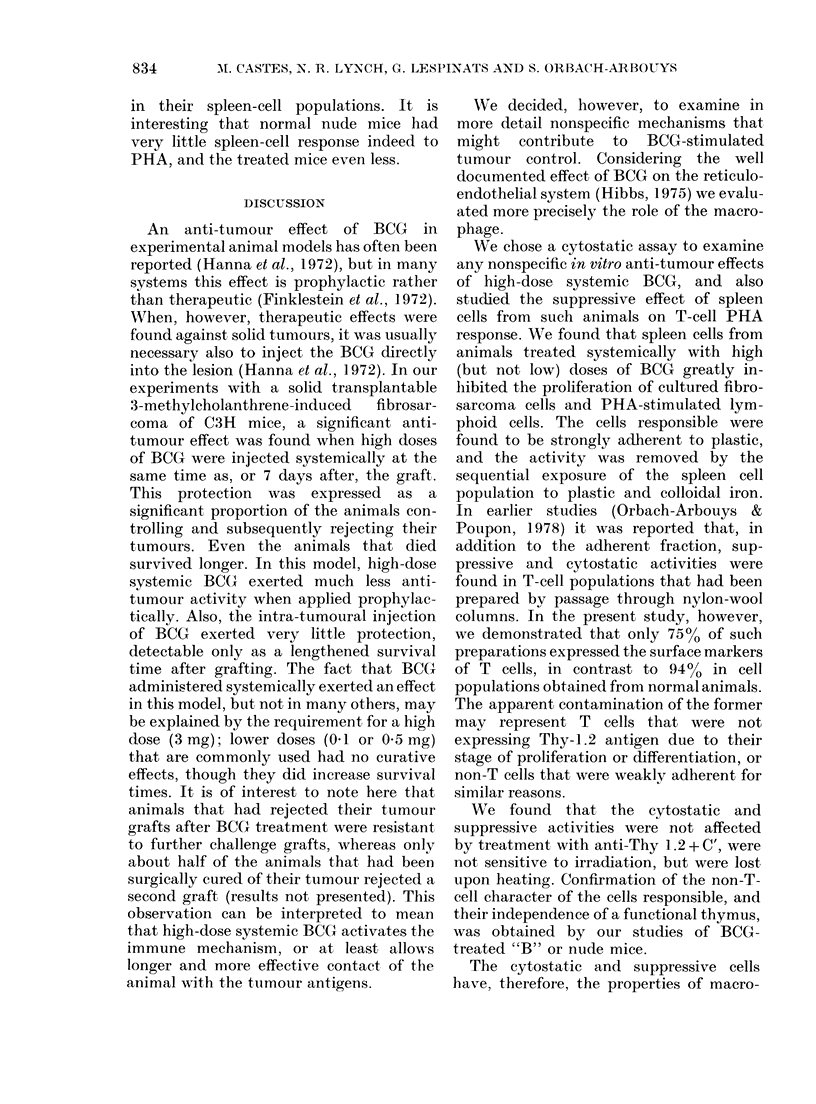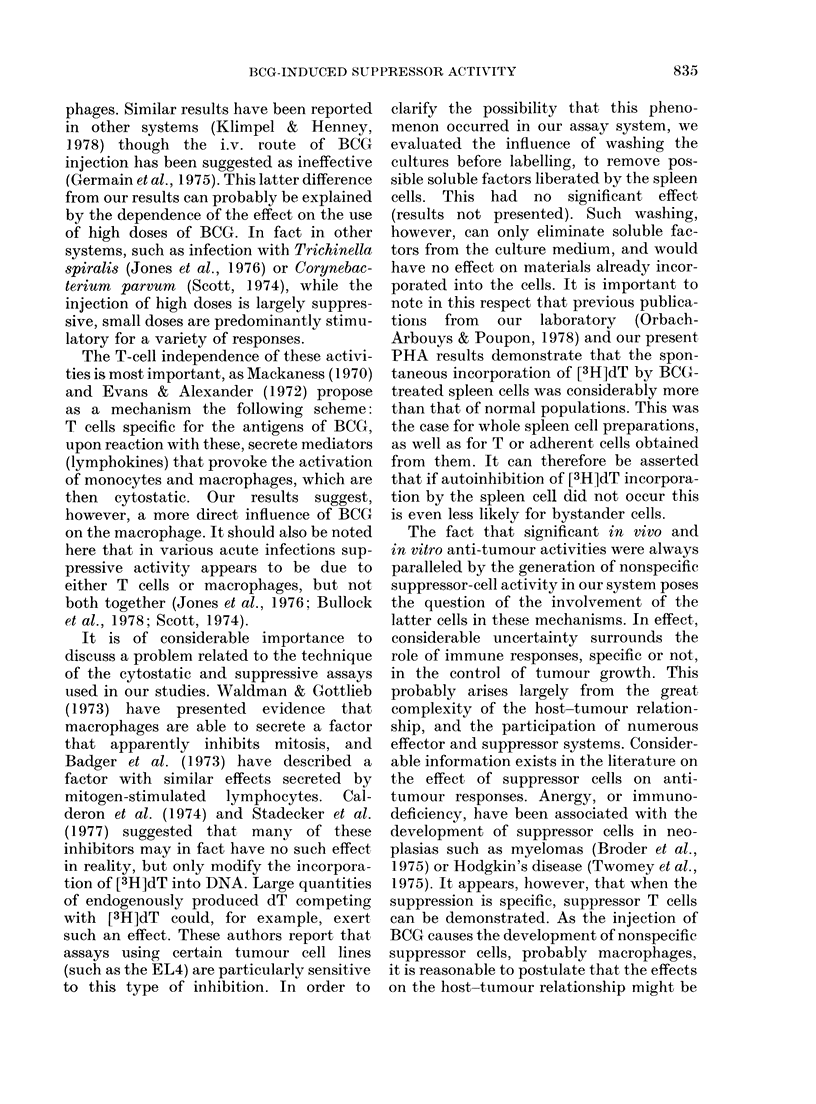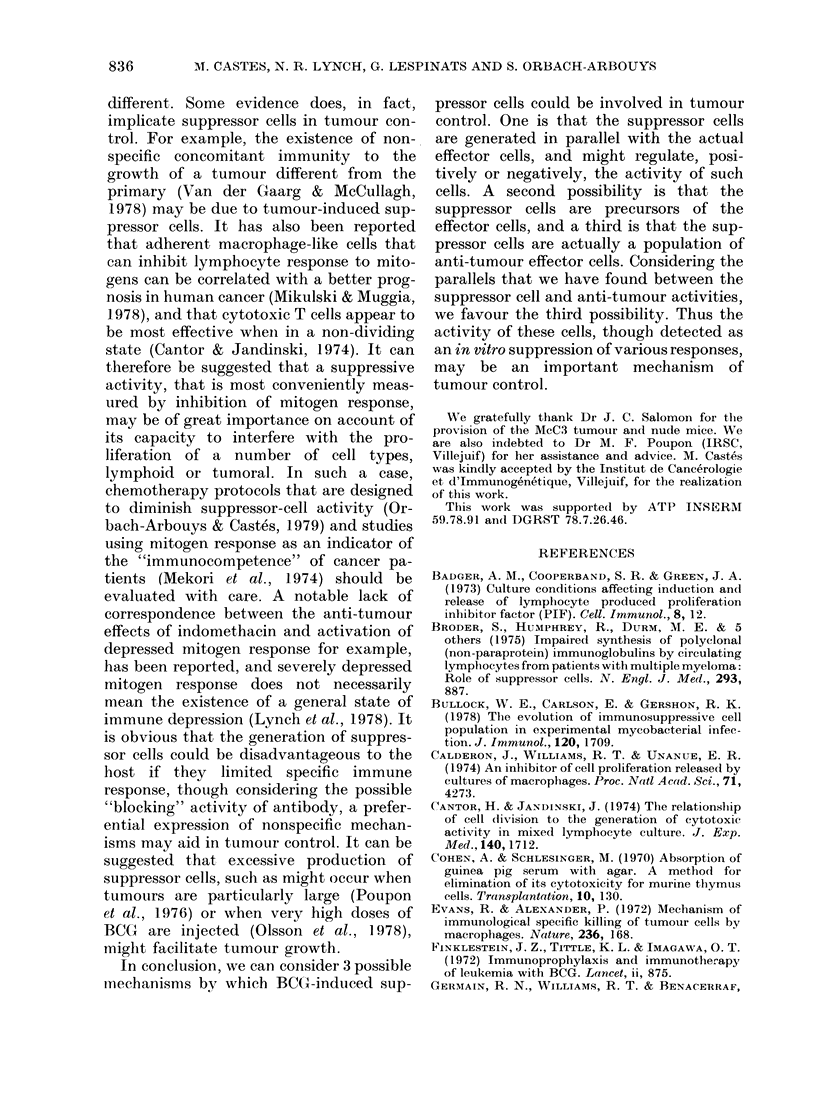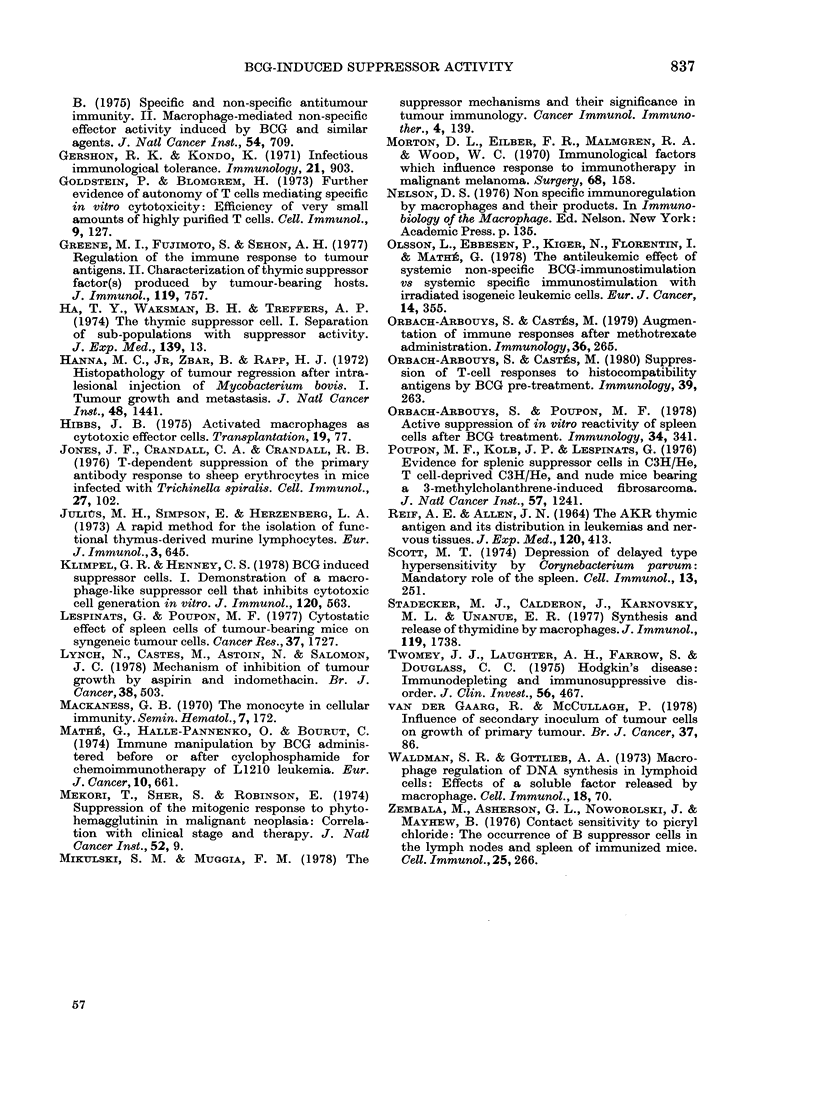# Possible role of macrophage-like suppressor cells in the anti-tumour activity of BCG.

**DOI:** 10.1038/bjc.1981.281

**Published:** 1981-12

**Authors:** M. Castés, N. R. Lynch, G. Lespinats, S. Orbach-Arbouys

## Abstract

The i.v. injection of high doses (3 mg) of BCG into C3H mice bearing a transplantable 3-methylcholanthrene-induced fibrosarcoma caused the regression of a significant proportion. This effect was most evident when the BCG was injected on the day of the graft, or 7 days later. The injection of this agent either 14 days before the graft, or in low doses (0.1 or 0.5 mg), or directly into the tumour (i.t.) only prolonged the survival of the animals. Spleen cells from systemic high-dose BCG-treated mice were found to exert a strong nonspecific cytostatic effect in vitro that was not an artefact of the test conditions, and was not expressed by cells from low-dose animals. The cytostatic effect was shown to be caused by cells with the characteristics of macrophages, i.e. they were strongly adherent, unaffected by treatment with anti-Thy 1.2 + C', radioresistant but heat-sensitive, and were detected in BCG-treated "B" mice. The spleens of high-dose BCG-treated mice also contained suppressor cells that were capable of inhibiting the in vitro reactivity of normal T cells to PHA. Like the cytostatic effect, this suppressor activity was not detected in low-dose mice, and the cells responsible had the properties of macrophages; the effect was lost after the removal of adherent cells by sequential exposure to plastic and colloidal iron, but was conserved after treatment with anti-Thy 1.2 + C'. T-cell-deprived animals, such as "B" or nude mice, also developed suppressor-cell activity when treated with systemic high-dose BCG. Close parallels became evident between the in vivo anti-tumour activity of BCG, the in vitro cytostatic effect, and the suppressor-cell activity. We here discuss the possible role of suppressor cells in the mechanism of action of this agent.


*Br. J. Cancer* (1981) **44**, 828

### POSSIBLE ROLE OF MACROPHAGE-LIKE SUPPRESSOR CELLS IN THE ANTI-TUMOUR ACTIVITY OF BCG

M. CASTÉS*, N. R. LYNCH†, G. LESPINATS† AND S. ORBACH-ARBOUYS‡

*From the *Escuela Vargas, Facultad de Medicina, Universidad Central de Venezuela, Caracas* 101, *Venezuela, the †Institut de Recherches Scientifiques sur le Cancer, and the ‡Institut de Cancérologie et d'Immunogénétique, Hôpital Paul-Brousse,* 94804 *Villejuif Cédex, France*

Received 14 May 1981 Accepted 18 August 1981

**Summary.—The i.v. injection of high doses (3 mg) of BCG into C3H mice bearing a transplantable 3-methylcholanthrene-induced fibrosarcoma caused the regression of a significant proportion. This effect was most evident when the BCG was injected on the day of the graft, or 7 days later. The injection of this agent either 14 days before the graft, or in low doses (0·1 or 0·5 mg), or directly into the tumour (i.t.) only prolonged the survival of the animals. Spleen cells from systemic high-dose BCG-treated mice were found to exert a strong nonspecific cytostatic effect *in vitro* that was not an artefact of the test conditions, and was not expressed by cells from low-dose animals. The cytostatic effect was shown to be caused by cells with the characteristics of macrophages, *i.e.* they were strongly adherent, unaffected by treatment with anti-Thy 1.2 + C′, radioresistant but heat-sensitive, and were detected in BCG-treated "B" mice.**

**The spleens of high-dose BCG-treated mice also contained suppressor cells that were capable of inhibiting the *in vitro* reactivity of normal T cells to PHA. Like the cytostatic effect, this suppressor activity was not detected in low-dose mice, and the cells responsible had the properties of macrophages; the effect was lost after the removal of adherent cells by sequential exposure to plastic and colloidal iron, but was conserved after treatment with anti-Thy 1.2 + C′. T-cell-deprived animals, such as "B" or nude mice, also developed suppressor-cell activity when treated with systemic high-dose BCG.**

**Close parallels became evident between the *in vivo* anti-tumour activity of BCG, the *in vitro* cytostatic effect, and the suppressor-cell activity. We here discuss the possible role of suppressor cells in the mechanism of action of this agent.**

A MAJOR ADVANCE in the field of immunology during the past few years has been the concept that immune reactions are controlled by negative feed-back mechanisms through suppressor cells (Gershon & Kondo, 1971). In many immunological model systems suppressor cells of different types, particularly suppressor T cells (Ha *et al.*, 1974), but also B cells (Zembala *et al.*, 1976) and macrophages (Nelson, 1976), have been implicated. There have been a number of reports that suppressor cells may also play a role in the immunosuppression found in some experimental tumour models (Greene *et al.*, 1977). In this respect, various bacterial vaccines such as BCG have been demonstrated to enhance nonspecifically a number of immune responses (Miller *et al.*, 1973) and are reported to produce a clinical improvement in some cases of leukaemia (Mathé *et al.*, 1974) and melanoma (Morton *et al.*, 1970). Also in various animal models, like the one described in

Correspondence to: Dr Simone Orbach-Arbouys, Institut de Cancérologie et d'Immunogénétique, Hôpital Paul-Brousse, 14 av. P.V. Couturier, 94804 Villejuif Cédex, France.

this report, BCG treatment can exert a marked curative effect (Hanna *et al.*, 1972). Studies in our laboratories have demonstrated that these agents can also induce nonspecific cell-mediated suppression of a variety of responses such as mitogen stimulation (Orbach-Arbouys & Poupon, 1978) or GvH and mixed lymphocyte reactions (Orbach-Arbouys & Castés, 1980).

In an attempt to elucidate the possible role of suppressor cells in the anti-tumour activity of BCG, we performed a series of experiments to correlate this effect with the *in vitro* cytostatic activity of various spleen-cell populations from BCG-treated mice. We demonstrated that systemic high-dose BCG exerted an anti-tumour effect *in vivo*, and induced the appearance of a cell population that was both cytostatic and suppressive for mitogen response. These cells were induced, and expressed their activity, in the absence of T cells, and had the properties of macrophages.

### MATERIALS AND METHODS

### *Mice*

6–8-week-old specific-pathogen-free C57-BL/6 mice were obtained from the breeding centre of the Centre National de la Recherche Scientifique, Orleans, France. C3H/He mice were provided by the Institut de Recherches Scientifiques sur le Cancer, Villejuif, France. Nude (*nu/nu*) mice were maintained in deliberately outbred colonies under barrier-protected conditions by Dr J. C. Salomon, IRSC, Villejuif, France. "B" mice were prepared by irradiation with 6 Gy, 1 week after thymectomy, and with 3 Gy a week later (A. J. S. Davies, personal communication). The mice were not reconstituted with marrow, as this can contain T cells. Such mice mounted no detectable plaque-forming cell (PFC) responses to injections of sheep erythrocytes, but did so against DNP-flagellin.

### *Tumours*

McC3-1 and McB6-1 were fibrosarcomas induced in C3H/He and C57BL/6 mice respectively by the i.m. injection of 1 mg 3-methylcholanthrene, and maintained by serial isogenic transplantation and freezing of various passages. Tumour grafts were performed ventrally by the s.c. introduction of small pieces ($\sim 1$ mm$^3$) of non-necrotic tissue *via* a trocar needle. The day of grafting was taken as Day 0 in all experiments.

### *BCG*

"Immuno BCG", which retains 95% viability after 3 months storage at 4°C, was kindly provided by the Pasteur Institute (Paris, France) and injected as described in the results. High doses (3 mg) i.v. caused marked splenomegaly and hepatomegaly, *viz.*, spleen weights of 180 and 450 mg on Days 7 and 14 respectively, and liver weights of 1·6 and 2·6 g on these days (controls: 95 mg and 1·3 g respectively).

### *Cell preparations*

*Spleen cells.*—Spleens from groups of 4 mice were aseptically removed, placed in Medium 199 (Eurobio, France) and gently squeezed between 2 sterile glass slides. The cell suspensions thus obtained were then filtered through gauze and washed with cold sterile Medium 199. For subsequent tests the suspensions were prepared in RPMI 1640 (Eurobio, France) supplemented with 2mM glutamine (Gibco, Grand Island, New York) and containing 5% fresh human AB serum (decomplemented by heating at 56°C for 30 min), 100 i.u./ml penicillin and 100 $\mu$g/ml streptomycin. When irradiated, the cells received 15 Gy delivered by a caesium bomb (Gravaton Industries, Gosport, Hampshire).

*Enriched T cells.*—T cells were isolated with nylon wool columns using the technique of Julius *et al.* (1973). Anti-Thy 1.2 serum (Lespinats & Poupon, 1977) plus guinea-pig complement killed 94% of the normal T-enriched suspension. This population is referred to throughout this report as nylon-purified T cells, or simply spleen T cells. Such preparations obtained from BCG-treated mice contained only 75% of cells killed by this treatment.

*Adherent spleen cells.*—$10^8$ spleen cells in 10 ml of RPMI with 5% inactivated foetal calf serum (Eurobio, France) were incubated horizontally in a 250ml plastic bottle (Falcon Plastics, California, U.S.A.); then after 60 min the supernatant was discarded and the cells adhering to the plastic were rinsed twice with PBS, incubated 3–10 min with

5 ml of Ca⁻ and Mg free Earle's medium (Gibco) containing 0·02% EDTA. The cells were harvested, washed and adjusted to a concentration of $5 \times 10^6$/ml.

*Depletion of adherent spleen cells using carbonyl-iron particles.*—$2 \times 10^7$ spleen cells in 20 ml of nutrient medium supplemented with 10% FCS were incubated at 37°C for 30 min with 50 mg of carbonyl-iron particles (particles E; GAF, Louvres, France) in a 250ml plastic bottle (Falcon Plastics). The cells adhering to the particles were magnetically removed by the procedure of Goldstein & Blomgren (1973). Treatment with anti-Thy 1.2 + C′ killed 3% of these cells.

*Depletion of Thy 1.2 cells.*—Anti-Thy 1.2 serum was prepared as described by Reif & Allen (1964). This antiserum killed 95% of normal thymocytes when incubated for 30 min at a dilution of 1:27 in the presence of guinea-pig complement. Lyophilized guinea-pig serum was obtained from Institut Pasteur Production, Paris, France, and was absorbed with agar as described by Cohen & Schlesinger (1970).

### In vitro *Culture techniques*

*Cytostatic test.*—Continuously cultivated McC3-1 and McB6-1 tumour cells, growing exponentially in RPMI 1640 supplemented with 5% inactivated foetal calf serum, were harvested by trypsinization and used as targets in cytostatic assays.

The method used was as described by Lespinats & Poupon (1977) with minor modifications. The tumour cells were washed and adjusted to $2 \times 10^4$ cells/ml in RPMI 1640 plus 2% inactivated foetal calf serum, 2mM glutamine and antibiotics. 100μl aliquots of the lymphocyte suspensions were added to 2500 tumour cells at ratios of 12·5:1, 25:1 and 50:1, in a total volume of 200 μl in microtitre plates (Falcon, 3040). All cultures were performed in triplicate. The microplates were covered and incubated at 37°C in an atmosphere of 5% $CO_2$ 95% air for 72 h, after which 1 μCi of [$^3$H]dT (TMM48, Commissariat de l'Energie Atomique, Saclay, France, sp. act. 27 Ci/mmol) was added to each well during the last 5 h of incubation. Cultures were harvested with a multiple automated sample harvester (MASH, Microbiological Associates, Bethesda, MA, U.S.A.) on glass-fibre filters (Reeve Angels, Clifton, N.Y.) which were placed in toluene plus Omnifluor (NEN, Dreieichhaim, West Germany) and counted in a Packard counter.

*Mitogen responsiveness.*—To test the responsiveness of spleen-cell populations to mitogens, 2·5 or $5 \times 10^5$ cells were placed in wells of Falcon 3040 microplates in a volume of 250 μl. The mitogen dose used was that found to be optimal for stimulation of normal spleen cells: 0·4 μg per well of PHA HA16 (Wellcome Research Laboratories, Beckenham, England).

To demonstrate the suppressive properties of cells from BCG-treated mice, $5 \times 10^5$ cells from normal or BCG-treated animals were cultivated with $2{\cdot}5 \times 10^5$ normal T cells and the same amount of PHA was added as previously. All cultures were performed in triplicate and incubated for 48 h in an atmosphere of 5% $CO_2$ in air. Five h before the end of the culture, 1 μCi of [$^3$H]dT was added to each well. The cells were harvested on glass-fibre filters, as described above, and the results expressed as mean ct/min of triplicate samples.

The suppressive effect of one population of spleen cells (B) against another (A) was calculated by the formula:

$$100 - 100 \times \frac{\text{ct/min (A+B) stimulated} - \text{ct/min (A+B) unstimulated}}{\text{ct/min (A) stimulated} - \text{ct/min (A) unstimulated}}$$

Preliminary studies demonstrated that the results of tests performed in this way were not influenced by factors such as cell crowding or nutritional alterations, and the optimal PHA concentration was not changed, even after BCG treatment.

*Statistical methods.*—The statistical significance of results obtained in survival experiments, cytostatic assays and mitogen stimulation were analysed by the $t$ test. The exact method of calculating significance by the $\chi^2$ test, using Yates' correction for small sample sizes, was also used in the analysis of survival data.

### RESULTS

### *Anti-tumour activity of BCG as measured* in vitro

BCG was injected at high (3 mg) or low (0·1 or 0·5 mg) i.v. doses into mice bearing the transplantable McC3-1 fibrosarcoma. The BCG was injected either on the day of the graft (Day 0), 14 days

TABLE I.—*Anti-tumour activity of BCG administered systemically against the McC3-1 tumour of C3H mice*

| BCG treatment | Survival | Survival time‡ (days) |
|---|---|---|
| None | 0/11 | 35·0 ± 4·4 |
| 3 mg, Day −14 | 2/7 | 61·8 ± 28·9** |
| 3 mg, Day 0† | 5/7** | {42, 106 |
| 3 mg, Day +7 | 5/10* | 59·7 ± 5·0** |
| 0·1 mg, Day 0 | 0/7 | 54·7 ± 9·2** |
| 0·5 mg, Day +7 | 0/7 | 52·8 ± 4·0** |
| 2 × 1·5 mg, Days 10 + 17§ | 0/9 | 52·3 ± 3·5** |

† Day 0 = Day of graft.

‡ Of the mice that died.

§ In this experiment the BCG was injected i.t., rather than i.v.

* $P = 0{\cdot}006$. ** $P \leqslant 0{\cdot}001$.

before (Day −14) or 7 days after (Day 7). In Table I, presenting the results of one of our experiments, it can be seen that all the untreated animals died about 35 days after grafting. When 3 mg BCG was injected on Day −14, only 2/7 of the tumours were rejected. Although this proportion was not statistically significant, the survival of the animals that died was significantly lengthened. When the high dose of BCG was injected on Day 0, 5/7 animals survived, this proportion being statistically significant, and the mean time of death of the remainder was considerably delayed. The injection of 3 mg BCG on Day 7 caused both a significant tumour rejection (5/10) and an increase in survival time of the mice that died. Low doses of BCG on Day 0 (0·1 mg) or Day 7 (0·5 mg) had no curative effect, though they did prolong life. It is of interest to note that when 1·5 mg of BCG was injected twice, with an interval of 7 days, into small growing tumours about 10 days after grafting (diameter ∼5 mm), there was no rejection in 9 mice, though the mean survival time was increased.

*Anti-tumour activity of BCG as measured* in vitro

A series of experiments was performed in which the cytostatic activity of different cell populations from normal and BCG-treated animals was measured on MC-induced C57Bl/6 fibrosarcoma cells cultivated *in vitro*. The BCG was administered i.v. at high dose (3 mg) and low (0·5 mg). This cytotoxicity was also tested for its sensitivity to irradiation (15 Gy) or heating (56°C, 30 min).

TABLE II.—In vitro *cytostatic effect of different cell populations from BCG-treated mice on McB6-1 tumour cells*

| Exp. | Spleen cells (50:1 tumour cell) | [$^3$H]-dT incorporation (ct/min) | % Inhibition by BCG |
|---|---|---|---|
| 1 | None | 29963 ± 2058 | |
| | Normal | 36131 ± 4082 | |
| | BCG (3 mg) | 8026 ± 629 | 78* |
| | Normal adherent | 19791 ± 4761 | |
| | BCG adherent | 3816 ± 642 | 81* |
| 2 | Normal | 265132 ± 30047 | |
| | BCG | 149300 ± 40583 | 44* |
| | Normal after anti Thy 1.2 + C′ | 235834 ± 25176 | |
| | BCG after anti Thy 1.2 + C | 100080 ± 35475 | 58* |
| 3 | Untreated "B" mice | 105532 ± 12011 | |
| | BCG "B" mice | 32135 ± 4135 | 69* |
| 4 | Normal | 124030 ± 30613 | |
| | BCG (0·5 mg) | 155938 ± 52210 | —NS |

* $P < 0{\cdot}001$.

Table II presents the results of 4 representative experiments, in which it can be seen (Exp. 1) that whole spleen-cell preparations from high-dose BCG animals inhibited the DNA synthesis of tumour cells with which they were co-cultivated *in vitro*.

This cytostatic effect was also exerted by the adherent cells from such populations, and treatment with anti-Thy 1.2 + C′ did not diminish their activity (Exp. 2).

It can also be seen (Exp. 3) that the spleen cells from high-dose BCG-treated "B" mice were cytostatic to the growth of McB6-1 tumour cells.

In contrast to these results, cells from low-dose BCG animals had no significant effect in these tests (Exp. 4).

The figure demonstrates that the cytostasis was not abolished when the whole (or adherent) spleen cells were irradiated (15 Gy) before culture. In contrast, heating at 56°C for 30 min eliminated this effect. No toxicity of the heated lymphoid-cell

FIGURE.—Effect of irradiation (15 Gy) or heating (56°C, 30 min) of spleen cells from control or BCG-treated (3 mg, i.v.) mice on [$^3$H]dT incorporation by cultured tumour cells.

preparations was detected, and heat-inactivated normal spleen cells had no inhibitory activity. In fact, increased DNA synthesis by the cultured tumour cells was found when heated cells were added, possibly because of nutritional factors. This effect was also noted when irradiated cells from normal animals were added to the cultures (Fig. 1).

*Responsiveness to mitogen*

It had been previously demonstrated in our laboratories that the i.v. injection of high doses of BCG into mice led to a series of modifications of the *in vitro* reactivity of lymphoid cells (Orbach-Arbouys & Poupon, 1978). We therefore extended our earlier studies of the development of cells capable of suppressing responses to PHA, and attempted to confirm their identity.

*Suppression by whole and adherent spleen-cell populations from BCG-treated mice of T-cell response to PHA*

To determine whether the depression of mitogen responsiveness of spleen cells from high-dose BCG mice was an active phenomenon, such cells from BCG-treated animals were co-cultivated with normal T cells, and the response of the mixed population to PHA was measured. It can be seen in Table III that the addition of unfractionated spleen cells from high-dose BCG mice diminished the response of the normal T cells to this mitogen.

In a similar manner, when adherent spleen cells from high-dose BCG mice were added to normal T cells, there was a severe depression of the response, whereas adherent cells from normal mice had virtually no effect.

This suppression persisted when the

TABLE III.—*Suppression of PHA responses by various spleen cell populations from BCG-treated mice*

| | Spleen cells (2:1 T cells) | +PHA | Added to normal T cells %PHA | Suppression % by BCG |
|---|---|---|---|---|
| Exp. 1 | None | — | 179195 ± 15234 | |
| | Normal † | 259162 ± 17251 | 123674 ± 17873 | |
| | BCG † | 1999 ± 1944 | 21691 ± 365 | 89·5** |
| | Adherent normal | 12721 ± 2052 | 151945 ± 10868 | |
| | Adherent BCG | 9244 ± 1832 | 5053 ± 605 | 96·7** |
| | Normal after anti Thy 1.2 + C′ | 13513 ± 1131 | 335266 ± 9389 | |
| | BCG after anti Thy 1.2 + C′ | 23766 ± 570 | 97151 ± 1271 | 74·5** |
| | BCG T cells (Fa-Fe)* | 141291 ± 33493 | 136459 ± 31273 | — NS |
| Exp. 2 | None | — | 199047 ± 9383 | |
| | Normal | 222801 ± 16072 | 176906 ± 59953 | |
| | Low-dose BCG | 97716 ± 16388 | 145026 ± 28887 | 23·4 NS |

† The spontaneous incorporation by normal spleen cells in Exp. 1 was 6268 ± 385 and of the BCG population 12600 ± 672.
* Fa-Fe: Falcon plastic-colloidal iron purified T cells. ** $P < 0{\cdot}001$.

TABLE IV.—*Suppression of PHA responses by high-dose BCG treated "B" or nude mouse spleen cell populations*

| Spleen cells | +PHA | Added to normal T cells† +PHA | % suppression by BCG |
|---|---|---|---|
| None | — | 64191 ± 5770 | |
| Untreated "B" mice | 15445 ± 8261* | 96181 ± 6180 | |
| BCG "B" mice | 12885 ± 1892* | 32379 ± 3468 | 69·8** |
| Untreated nude mice | 43497 ± 208* | 74635 ± 273 | |
| BCG nude mice | 10562 ± 102 | 27283 ± 1650 | 57·7** |

* Background of untreated "B" mice was: 26699 ± 13694 and of nude mice: 30647 ± 175. ** $P \leqslant 0{\cdot}001$.

spleen cells were treated with anti-Thy 1.2 serum + C′. Alternatively, when highly purified T-cell preparations were obtained by the removal of cells adhering to plastic and colloid iron, no suppression was observed. It should be noted that T cells prepared by passage through nylon columns were previously found to show some suppressive effects (Orbach-Arbouys & Poupon, 1978), possibly owing to the presence of some residual adherent cells.

It can also be seen in Table III that mice treated with low doses of BCG (0·5 mg) did not develop significant suppressive activity, whether the BCG was injected 14 or 21 days before the experiment. When the spleen cells of such mice were tested 6 weeks after injection, a low but significant (38%, $P < 0{\cdot}01$) suppression was detected (results not presented).

It is of interest that the spontaneous [$^3$H]dT incorporation by normal spleen cells was significantly lower than that of the BCG population in all our experiments.

*Suppression of T-cell PHA responses by spleen cells from BCG-treated B and nude mice*

In the light of the previous results, it was thought important to determine whether in fact T cells were necessary for the generation or expression of the suppressive effect on PHA responsiveness.

In Table IV it can be seen that B mice, that effectively lacked PHA responses, when treated with high doses of BCG developed cells in their spleens that were capable of suppressing the PHA response of normal T cells. Similarly, BCG-treated nude mice developed suppressive activity in their spleen-cell populations. It is interesting that normal nude mice had very little spleen-cell response indeed to PHA, and the treated mice even less.

### DISCUSSION

An anti-tumour effect of BCG in experimental animal models has often been reported (Hanna *et al.*, 1972), but in many systems this effect is prophylactic rather than therapeutic (Finklestein *et al.*, 1972). When, however, therapeutic effects were found against solid tumours, it was usually necessary also to inject the BCG directly into the lesion (Hanna *et al.*, 1972). In our experiments with a solid transplantable 3-methylcholanthrene-induced fibrosarcoma of C3H mice, a significant anti-tumour effect was found when high doses of BCG were injected systemically at the same time as, or 7 days after, the graft. This protection was expressed as a significant proportion of the animals controlling and subsequently rejecting their tumours. Even the animals that died survived longer. In this model, high-dose systemic BCG exerted much less anti-tumour activity when applied prophylactically. Also, the intra-tumoural injection of BCG exerted very little protection, detectable only as a lengthened survival time after grafting. The fact that BCG administered systemically exerted an effect in this model, but not in many others, may be explained by the requirement for a high dose (3 mg); lower doses (0·1 or 0·5 mg) that are commonly used had no curative effects, though they did increase survival times. It is of interest to note here that animals that had rejected their tumour grafts after BCG treatment were resistant to further challenge grafts, whereas only about half of the animals that had been surgically cured of their tumour rejected a second graft (results not presented). This observation can be interpreted to mean that high-dose systemic BCG activates the immune mechanism, or at least allows longer and more effective contact of the animal with the tumour antigens.

We decided, however, to examine in more detail nonspecific mechanisms that might contribute to BCG-stimulated tumour control. Considering the well documented effect of BCG on the reticuloendothelial system (Hibbs, 1975) we evaluated more precisely the role of the macrophage.

We chose a cytostatic assay to examine any nonspecific *in vitro* anti-tumour effects of high-dose systemic BCG, and also studied the suppressive effect of spleen cells from such animals on T-cell PHA response. We found that spleen cells from animals treated systemically with high (but not low) doses of BCG greatly inhibited the proliferation of cultured fibrosarcoma cells and PHA-stimulated lymphoid cells. The cells responsible were found to be strongly adherent to plastic, and the activity was removed by the sequential exposure of the spleen cell population to plastic and colloidal iron. In earlier studies (Orbach-Arbouys & Poupon, 1978) it was reported that, in addition to the adherent fraction, suppressive and cytostatic activities were found in T-cell populations that had been prepared by passage through nylon-wool columns. In the present study, however, we demonstrated that only 75% of such preparations expressed the surface markers of T cells, in contrast to 94% in cell populations obtained from normal animals. The apparent contamination of the former may represent T cells that were not expressing Thy-1.2 antigen due to their stage of proliferation or differentiation, or non-T cells that were weakly adherent for similar reasons.

We found that the cytostatic and suppressive activities were not affected by treatment with anti-Thy $1.2+C'$, were not sensitive to irradiation, but were lost upon heating. Confirmation of the non-T-cell character of the cells responsible, and their independence of a functional thymus, was obtained by our studies of BCG-treated "B" or nude mice.

The cytostatic and suppressive cells have, therefore, the properties of macrophages. Similar results have been reported in other systems (Klimpel & Henney, 1978) though the i.v. route of BCG injection has been suggested as ineffective (Germain *et al.*, 1975). This latter difference from our results can probably be explained by the dependence of the effect on the use of high doses of BCG. In fact in other systems, such as infection with *Trichinella spiralis* (Jones *et al.*, 1976) or *Corynebacterium parvum* (Scott, 1974), while the injection of high doses is largely suppressive, small doses are predominantly stimulatory for a variety of responses.

The T-cell independence of these activities is most important, as Mackaness (1970) and Evans & Alexander (1972) propose as a mechanism the following scheme: T cells specific for the antigens of BCG, upon reaction with these, secrete mediators (lymphokines) that provoke the activation of monocytes and macrophages, which are then cytostatic. Our results suggest, however, a more direct influence of BCG on the macrophage. It should also be noted here that in various acute infections suppressive activity appears to be due to either T cells or macrophages, but not both together (Jones *et al.*, 1976; Bullock *et al.*, 1978; Scott, 1974).

It is of considerable importance to discuss a problem related to the technique of the cytostatic and suppressive assays used in our studies. Waldman & Gottlieb (1973) have presented evidence that macrophages are able to secrete a factor that apparently inhibits mitosis, and Badger *et al.* (1973) have described a factor with similar effects secreted by mitogen-stimulated lymphocytes. Calderon *et al.* (1974) and Stadecker *et al.* (1977) suggested that many of these inhibitors may in fact have no such effect in reality, but only modify the incorporation of [$^3$H]dT into DNA. Large quantities of endogenously produced dT competing with [$^3$H]dT could, for example, exert such an effect. These authors report that assays using certain tumour cell lines (such as the EL4) are particularly sensitive to this type of inhibition. In order to clarify the possibility that this phenomenon occurred in our assay system, we evaluated the influence of washing the cultures before labelling, to remove possible soluble factors liberated by the spleen cells. This had no significant effect (results not presented). Such washing, however, can only eliminate soluble factors from the culture medium, and would have no effect on materials already incorporated into the cells. It is important to note in this respect that previous publications from our laboratory (Orbach-Arbouys & Poupon, 1978) and our present PHA results demonstrate that the spontaneous incorporation of [$^3$H]dT by BCG-treated spleen cells was considerably more than that of normal populations. This was the case for whole spleen cell preparations, as well as for T or adherent cells obtained from them. It can therefore be asserted that if autoinhibition of [$^3$H]dT incorporation by the spleen cell did not occur this is even less likely for bystander cells.

The fact that significant *in vivo* and *in vitro* anti-tumour activities were always paralleled by the generation of nonspecific suppressor-cell activity in our system poses the question of the involvement of the latter cells in these mechanisms. In effect, considerable uncertainty surrounds the role of immune responses, specific or not, in the control of tumour growth. This probably arises largely from the great complexity of the host–tumour relationship, and the participation of numerous effector and suppressor systems. Considerable information exists in the literature on the effect of suppressor cells on anti-tumour responses. Anergy, or immunodeficiency, have been associated with the development of suppressor cells in neoplasias such as myelomas (Broder *et al.*, 1975) or Hodgkin's disease (Twomey *et al.*, 1975). It appears, however, that when the suppression is specific, suppressor T cells can be demonstrated. As the injection of BCG causes the development of nonspecific suppressor cells, probably macrophages, it is reasonable to postulate that the effects on the host–tumour relationship might be different. Some evidence does, in fact, implicate suppressor cells in tumour control. For example, the existence of non-specific concomitant immunity to the growth of a tumour different from the primary (Van der Gaarg & McCullagh, 1978) may be due to tumour-induced suppressor cells. It has also been reported that adherent macrophage-like cells that can inhibit lymphocyte response to mitogens can be correlated with a better prognosis in human cancer (Mikulski & Muggia, 1978), and that cytotoxic T cells appear to be most effective when in a non-dividing state (Cantor & Jandinski, 1974). It can therefore be suggested that a suppressive activity, that is most conveniently measured by inhibition of mitogen response, may be of great importance on account of its capacity to interfere with the proliferation of a number of cell types, lymphoid or tumoral. In such a case, chemotherapy protocols that are designed to diminish suppressor-cell activity (Orbach-Arbouys & Castés, 1979) and studies using mitogen response as an indicator of the "immunocompetence" of cancer patients (Mekori *et al.*, 1974) should be evaluated with care. A notable lack of correspondence between the anti-tumour effects of indomethacin and activation of depressed mitogen response for example, has been reported, and severely depressed mitogen response does not necessarily mean the existence of a general state of immune depression (Lynch *et al.*, 1978). It is obvious that the generation of suppressor cells could be disadvantageous to the host if they limited specific immune response, though considering the possible "blocking" activity of antibody, a preferential expression of nonspecific mechanisms may aid in tumour control. It can be suggested that excessive production of suppressor cells, such as might occur when tumours are particularly large (Poupon *et al.*, 1976) or when very high doses of BCG are injected (Olsson *et al.*, 1978), might facilitate tumour growth.

In conclusion, we can consider 3 possible mechanisms by which BCG-induced suppressor cells could be involved in tumour control. One is that the suppressor cells are generated in parallel with the actual effector cells, and might regulate, positively or negatively, the activity of such cells. A second possibility is that the suppressor cells are precursors of the effector cells, and a third is that the suppressor cells are actually a population of anti-tumour effector cells. Considering the parallels that we have found between the suppressor cell and anti-tumour activities, we favour the third possibility. Thus the activity of these cells, though detected as an *in vitro* suppression of various responses, may be an important mechanism of tumour control.

We gratefully thank Dr J. C. Salomon for the provision of the McC3 tumour and nude mice. We are also indebted to Dr M. F. Poupon (IRSC, Villejuif) for her assistance and advice. M. Castés was kindly accepted by the Institut de Cancérologie et d'Immunogénétique, Villejuif, for the realization of this work.

This work was supported by ATP INSERM 59.78.91 and DGRST 78.7.26.46.

### REFERENCES

BADGER, A. M., COOPERBAND, S. R. & GREEN, J. A. (1973) Culture conditions affecting induction and release of lymphocyte produced proliferation inhibitor factor (PIF). *Cell. Immunol.*, **8**, 12.

BRODER, S., HUMPHREY, R., DURM, M. E. & 5 others (1975) Impaired synthesis of polyclonal (non-paraprotein) immunoglobulins by circulating lymphocytes from patients with multiple myeloma: Role of suppressor cells. *N. Engl. J. Med.*, **293**, 887.

BULLOCK, W. E., CARLSON, E. & GERSHON, R. K. (1978) The evolution of immunosuppressive cell population in experimental mycobacterial infection. *J. Immunol.*, **120**, 1709.

CALDERON, J., WILLIAMS, R. T. & UNANUE, E. R. (1974) An inhibitor of cell proliferation released by cultures of macrophages. *Proc. Natl Acad. Sci.*, **71**, 4273.

CANTOR, H. & JANDINSKI, J. (1974) The relationship of cell division to the generation of cytotoxic activity in mixed lymphocyte culture. *J. Exp. Med.*, **140**, 1712.

COHEN, A. & SCHLESINGER, M. (1970) Absorption of guinea pig serum with agar. A method for elimination of its cytotoxicity for murine thymus cells. *Transplantation*, **10**, 130.

EVANS, R. & ALEXANDER, P. (1972) Mechanism of immunological specific killing of tumour cells by macrophages. *Nature*, **236**, 168.

FINKLESTEIN, J. Z., TITTLE, K. L. & IMAGAWA, O. T. (1972) Immunoprophylaxis and immunotherapy of leukemia with BCG. *Lancet*, ii, 875.

GERMAIN, R. N., WILLIAMS, R. T. & BENACERRAF,

B. (1975) Specific and non-specific antitumour immunity. II. Macrophage-mediated non-specific effector activity induced by BCG and similar agents. *J. Natl Cancer Inst.*, **54**, 709.

GERSHON, R. K. & KONDO, K. (1971) Infectious immunological tolerance. *Immunology*, **21**, 903.

GOLDSTEIN, P. & BLOMGREM, H. (1973) Further evidence of autonomy of T cells mediating specific *in vitro* cytotoxicity: Efficiency of very small amounts of highly purified T cells. *Cell. Immunol.*, **9**, 127.

GREENE, M. I., FUJIMOTO, S. & SEHON, A. H. (1977) Regulation of the immune response to tumour antigens. II. Characterization of thymic suppressor factor(s) produced by tumour-bearing hosts. *J. Immunol.*, **119**, 757.

HA, T. Y., WAKSMAN, B. H. & TREFFERS, A. P. (1974) The thymic suppressor cell. I. Separation of sub-populations with suppressor activity. *J. Exp. Med.*, **139**, 13.

HANNA, M. C., JR, ZBAR, B. & RAPP, H. J. (1972) Histopathology of tumour regression after intralesional injection of *Mycobacterium bovis*. I. Tumour growth and metastasis. *J. Natl Cancer Inst.*, **48**, 1441.

HIBBS, J. B. (1975) Activated macrophages as cytotoxic effector cells. *Transplantation*, **19**, 77.

JONES, J. F., CRANDALL, C. A. & CRANDALL, R. B. (1976) T-dependent suppression of the primary antibody response to sheep erythrocytes in mice infected with *Trichinella spiralis*. *Cell. Immunol.*, **27**, 102.

JULIUS, M. H., SIMPSON, E. & HERZENBERG, L. A. (1973) A rapid method for the isolation of functional thymus-derived murine lymphocytes. *Eur. J. Immunol.*, **3**, 645.

KLIMPEL, G. R. & HENNEY, C. S. (1978) BCG induced suppressor cells. I. Demonstration of a macrophage-like suppressor cell that inhibits cytotoxic cell generation *in vitro*. *J. Immunol.*, **120**, 563.

LESPINATS, G. & POUPON, M. F. (1977) Cytostatic effect of spleen cells of tumour-bearing mice on syngeneic tumour cells. *Cancer Res.*, **37**, 1727.

LYNCH, N., CASTES, M., ASTOIN, N. & SALOMON, J. C. (1978) Mechanism of inhibition of tumour growth by aspirin and indomethacin. *Br. J. Cancer*, **38**, 503.

MACKANESS, G. B. (1970) The monocyte in cellular immunity. *Semin. Hematol.*, **7**, 172.

MATHÉ, G., HALLE-PANNENKO, O. & BOURUT, C. (1974) Immune manipulation by BCG administered before or after cyclophosphamide for chemoimmunotherapy of L1210 leukemia. *Eur. J. Cancer*, **10**, 661.

MEKORI, T., SHER, S. & ROBINSON, E. (1974) Suppression of the mitogenic response to phytohemagglutinin in malignant neoplasia: Correlation with clinical stage and therapy. *J. Natl Cancer Inst.*, **52**, 9.

MIKULSKI, S. M. & MUGGIA, F. M. (1978) The suppressor mechanisms and their significance in tumour immunology. *Cancer Immunol. Immunother.*, **4**, 139.

MORTON, D. L., EILBER, F. R., MALMGREN, R. A. & WOOD, W. C. (1970) Immunological factors which influence response to immunotherapy in malignant melanoma. *Surgery*, **68**, 158.

NELSON, D. S. (1976) Non specific immunoregulation by macrophages and their products. In *Immunobiology of the Macrophage*. Ed. Nelson. New York: Academic Press. p. 135.

OLSSON, L., EBBESEN, P., KIGER, N., FLORENTIN, I. & MATHÉ, G. (1978) The antileukemic effect of systemic non-specific BCG-immunostimulation *vs* systemic specific immunostimulation with irradiated isogeneic leukemic cells. *Eur. J. Cancer*, **14**, 355.

ORBACH-ARBOUYS, S. & CASTÉS, M. (1979) Augmentation of immune responses after methotrexate administration. *Immunology*, **36**, 265.

ORBACH-ARBOUYS, S. & CASTÉS, M. (1980) Suppression of T-cell responses to histocompatibility antigens by BCG pre-treatment. *Immunology*, **39**, 263.

ORBACH-ARBOUYS, S. & POUPON, M. F. (1978) Active suppression of *in vitro* reactivity of spleen cells after BCG treatment. *Immunology*, **34**, 341.

POUPON, M. F., KOLB, J. P. & LESPINATS, G. (1976) Evidence for splenic suppressor cells in C3H/He, T cell-deprived C3H/He, and nude mice bearing a 3-methylcholanthrene-induced fibrosarcoma. *J. Natl Cancer Inst.*, **57**, 1241.

REIF, A. E. & ALLEN, J. N. (1964) The AKR thymic antigen and its distribution in leukemias and nervous tissues. *J. Exp. Med.*, **120**, 413.

SCOTT, M. T. (1974) Depression of delayed type hypersensitivity by *Corynebacterium parvum*: Mandatory role of the spleen. *Cell. Immunol.*, **13**, 251.

STADECKER, M. J., CALDERON, J., KARNOVSKY, M. L. & UNANUE, E. R. (1977) Synthesis and release of thymidine by macrophages. *J. Immunol.*, **119**, 1738.

TWOMEY, J. J., LAUGHTER, A. H., FARROW, S. & DOUGLASS, C. C. (1975) Hodgkin's disease: Immunodepleting and immunosuppressive disorder. *J. Clin. Invest.*, **56**, 467.

VAN DER GAARG, R. & MCCULLAGH, P. (1978) Influence of secondary inoculum of tumour cells on growth of primary tumour. *Br. J. Cancer*, **37**, 86.

WALDMAN, S. R. & GOTTLIEB, A. A. (1973) Macrophage regulation of DNA synthesis in lymphoid cells: Effects of a soluble factor released by macrophage. *Cell. Immunol.*, **18**, 70.

ZEMBALA, M., ASHERSON, G. L., NOWOROLSKI, J. & MAYHEW, B. (1976) Contact sensitivity to picryl chloride: The occurrence of B suppressor cells in the lymph nodes and spleen of immunized mice. *Cell. Immunol.*, **25**, 266.